# The Up-to-date Treatment for Diabetes and Prevention of its Complications

**DOI:** 10.14789/ejmj.JMJ24-0030-R

**Published:** 2024-12-31

**Authors:** YUYA NISHIDA, HIROTAKA WATADA

**Affiliations:** 1 Department of Metabolism & Endocrinology, Juntendo University Graduate School of Medicine, Tokyo, Japan; 1 Department of Metabolism & Endocrinology, Juntendo University Graduate School of Medicine, Tokyo, Japan

**Keywords:** diabetes, insulin, advocacy, stigma, autophagy

## Abstract

Diabetes mellitus, characterized by high blood glucose due to inadequate insulin action, comprises two main types: type 1, an autoimmune disease, and type 2, marked by insulin resistance. This review provides a comprehensive overview of diabetes management and treatment advancements. Effective diabetes management includes maintaining blood glucose levels within normal ranges and monitoring HbA1c, a marker reflecting average glucose levels over the past few months. Historically, the discovery of insulin in 1921 revolutionized diabetes treatment, significantly extending patient life expectancy. Current treatment strategies encompass diet, exercise, and pharmacotherapy. The diet involves a balanced intake of carbohydrates, proteins, and fats, while exercise, including aerobic and resistance training, improves insulin sensitivity and glucose control. Pharmacotherapy options include insulin therapy and oral hypoglycemic agents, like metformin and empagliflozin, each with specific mechanisms of action. Innovative treatments include SGLT2 inhibitors and GLP-1 receptor agonists, which aid in glucose control and offer additional benefits like weight loss and improved cardiovascular outcomes. Continuous glucose monitoring (CGM) and insulin pumps represent technological advancements enhancing glycemic control through real-time monitoring and automated insulin delivery. We must pay attention to diabetes-related stigma, which we should overcome by advocacy. The diabetes education programs at Juntendo University Hospital aim to improve patient self-management through comprehensive diet, exercise, and medication education. We emphasize the importance of integrating the latest research and societal support to enable diabetic patients to lead healthy, fulfilling lives.

## Introduction

Diabetes mellitus is a chronic disease that continues to increase worldwide and requires proper management and treatment. This review includes a general description of diabetes, its history, the basics of treatment, the latest treatment techniques, treatment goals, overcoming stigma, the importance of basic research, and diabetes education programs in our department at Juntendo University Hospital.

**Figure 1:**
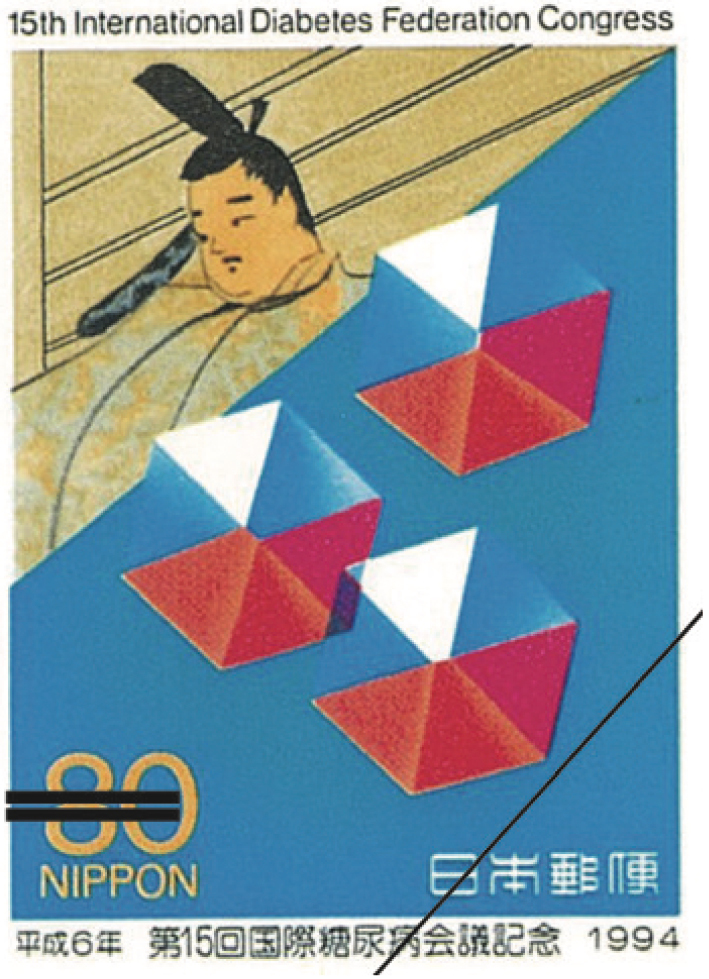
A commemorative stamp for the 15^th^ International Diabetes Federation Congress. Images of Fujiwara-no-Michinaga and insulin crystals are shown

As an introduction, the picture of Fujiwara-no- Michinaga (966-1028), a well-known statesman in the Heian era in ancient Japan, was shown in the seminar (Figure 1). According to his diary and his colleagues' records, he is supposed to be the first diabetic patient recorded in Japanese history, as he suffered from various complications related to diabetes, such as thirst, blindness, and chest pain.

## Basics of diabetes mellitus

### What is diabetes?

Diabetes mellitus is a condition in which blood glucose levels are chronically high due to inadequate action of the hormone insulin in the body^1)^. Insulin is secreted by the beta cells of the pancreas and plays an important role in lowering blood glucose levels. It is the only hormone that lowers blood glucose levels and is essential for incorporating glucose into the cells, using it as energy, or storing the excess glucose as lipids.

### Types of diabetes

**Figure 2:**
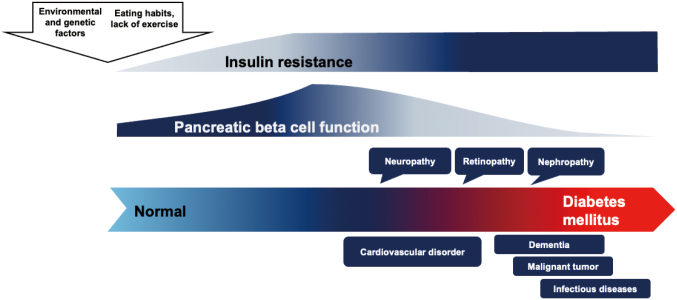
History of type 2 diabetes. Insulin resistance followed by pancreatic beta cell failure contributes to the development of type 2 diabetes.

There are two main types of diabetes: type 1 diabetes and type 2 diabetes. Type 1 diabetes is an autoimmune disease in which the body does not produce any insulin because the beta cells that secrete insulin are destroyed by autoimmunity. Type 2 diabetes, on the other hand, occurs because of an increase in insulin resistance, which means that insulin becomes less effective. With insulin resistance, pancreatic beta cells try to compensate for it. However, exhausted beta cells eventually secrete insufficient amounts of insulin. Type 2 diabetes is greatly influenced by genetic factors and lifestyle habits, such as diet and lack of exercise (Figure 2)^2)^.

### Blood glucose management

Blood glucose control is very important in the treatment of diabetes. Normal blood glucose levels are tightly controlled, approximately 70-100 mg/dL at fasting and 140 mg/dL or less two hours after a meal. Diabetic patients often exceed these ranges, so management of blood glucose levels is especially important. Proper management of blood glucose levels can prevent complications of diabetes and improve quality of life.

### Importance of HbA1c

HbA1c (glycohemoglobin) is a value that reflects average blood glucose levels over the past month or two; a high HbA1c indicates persistently high blood glucose levels and an increased risk of complications. A healthy person's HbA1c is about 6% or less, while diabetic patients generally aim for 7% or less to prevent complications^3)^.

### Symptoms in diabetes

High blood glucose provokes symptoms of diabetes, such as thirst, polydipsia, polyuria, and weight loss. However, these symptoms often go unnoticed until the disease progresses, so regular physical examinations are important. Prolonged high blood glucose can lead to complications such as neuropathy (for example numbness), retinopathy (vision loss), and nephropathy (kidney failure).

## History of treatment for diabetes

### Before the discovery of insulin

The treatment of diabetes changed dramatically before and after the discovery of insulin in 1921. Before the discovery of insulin, patients with type 1 diabetes suffered weight loss due to polyuria and polydipsia associated with high blood glucose and just waited for death with no cure. The average life expectancy of diabetics in this era was very short, and many patients with type 1 diabetes died at a young age.

### Discovery of insulin

**Figure 3:**
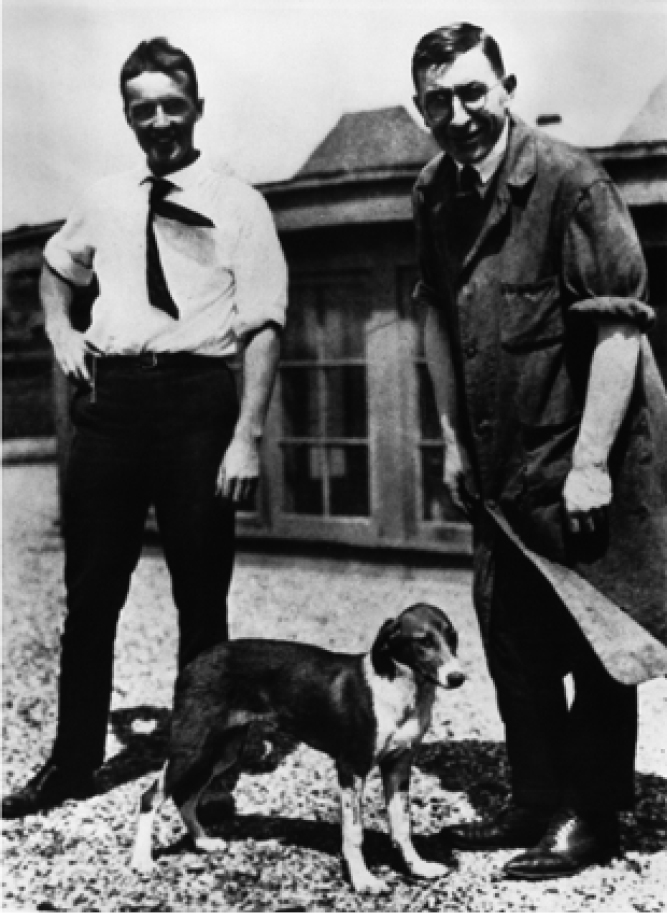
An image of Frederick Banting (right) & Charles Best, with a dog after pancreatectomy rescued by insulin injection From the website of Science History Institute Museum & Library (https://www.sciencehistory.org)

In 1921, Frederick Banting, a Canadian surgeon and Charles Best, his student, discovered insulin (Figure 3)^4)^. This discovery revolutionized the treatment of diabetes, and many patients' lives were saved by insulin therapy. After the discovery of insulin, it became widely used to treat diabetics. Initially, insulin extracted from cattle and pigs was used, but later human insulin was mass-produced using genetic modification technology. This has stabilized the supply of insulin and made it possible for more patients to receive treatment. The discovery of insulin dramatically increased the life expectancy of diabetic patients, and prevention of complications became the next challenge.

### Basics of diabetes treatment

#### Diet therapy

Treatment of diabetes consists of three main components: medication, exercise, and diet. Diet therapy is important to maintain adequate caloric intake and nutritional balance. It is recommended that meal plans be based on target body weight and energy expenditure, which varies from patient to patient. An appropriate balance of carbohydrates, protein, and lipids should be maintained. However, a proper balance between those nutrients is still in debate^5)^. Fiber intake and sodium restriction are also important in the diabetic's diet. Salt intake should be reduced to prevent hypertension and decrease the risk of atherosclerosis. Healthy fat choices and fiber-rich foods are also recommended.

#### Dietary examples

For example, a 50-year-old male office worker, 170 cm tall and 70 kg in weight, would set his target weight at 64 kg. 2000 kcal per day is appropriate for his daily energy intake, depending on his activity level. This energy is divided into three meals, and a well-balanced diet is maintained. Looking at the example dietary menu for the patients, we would be surprised to know how much he can eat, which must be more than we expected.

### Therapeutic exercise

Exercise therapy is one of the basic pillars of diabetes treatment. Exercise improves the effectiveness of insulin and lowers blood glucose levels. A combination of aerobic exercise (aerobics) and resistance exercise (strength training) is considered effective. Aerobic exercise uses oxygen to burn fat and sugar and increases endurance. Walking, jogging, cycling, and swimming are typical examples. Resistance exercise, on the other hand, is muscle-building exercise that increases muscle mass and raises basal metabolism. Strength training and weightlifting fall under this category.

### Specific examples and benefits of exercise

We present the results of a study conducted by Juntendo University regarding exercise therapy^6)^. The study confirmed that when patients with type 2 diabetes in hospital adopt exercise therapy, the fat stored in muscles is reduced, and insulin efficacy is improved. Specifically, the group that combined diet and exercise therapy had improved insulin sensitivity and better control of blood glucose levels compared to the diet-only group.

### Recommended amount of exercise

Realistically, it is recommended to exercise at least three times a week for 15 to 30 minutes each time. However, since it is sometimes difficult to take up a significant amount of time in our busy daily lives, it is important to continue to exercise, even for short periods of time. Continuity is strength, and daily exercise is essential for diabetes treatment.

### Pharmacotherapy for diabetes

It must be noted that the guidelines for pharmacotherapy of diabetes are established based on evidence-based medicine^7)^. One of the most reliable evaluations of the effects of pharmacotherapy is a double-blind, randomized clinical trial. We know that specialist opinions are sometimes important. However, their evidence level is limited. Pharmacotherapy for diabetes treatment includes insulin therapy and oral hypoglycemic agents. The following details the major types of drug therapy and their roles. In the seminar, several representative clinical studies such as DCCT for type 1 diabetes (Diabetes Control and Complications Trial)^8)^ and EMPA-REG for type 2 diabetes (Empagliflozin Cardiovascular Outcome Event Trial in Type 2 diabetes Mellitus Patients)^9)^ are shown.

#### Oral hypoglycemic drug

Oral hypoglycemic drugs are used primarily in patients with type 2 diabetes. Below are the major types of medications available.

**1. Sulfonylureas (SU drugs):** drugs that stimulate insulin secretion. An example is glimepiride.

**2. Biguanides:** suppress gluconeogenesis in the liver and improve insulin sensitivity. A typical example is metformin, which is one of the most widely used due to its safety and inexpensiveness.

**3. Thiazolidinediones:** drugs that increase insulin sensitivity. Pioglitazone is a well-known example.

**4. DPP-4 inhibitors:** these drugs stimulate insulin secretion and inhibit glucagon secretion. Examples include sitagliptin, which is more frequently prescribed in Japan than in other countries.

**5. SGLT2 inhibitors:** These drugs inhibit sugar reabsorption in the kidneys and cause sugar to be excreted in the urine. Empagliflozin, whose efficiency was proved in the EMPA-REG trial, is one example.

**6. GLP-1 receptor agonist:** GLP-1 receptor agonists promote postprandial insulin secretion and suppress appetite, reducing body weight. Previously used in subcutaneous injection, now available as an oral drug.

### Insulin therapy

Insulin therapy is considered when the body does not produce enough insulin. It is mandatory to use insulin in patients with type 1 diabetes, and in patients with type 2 diabetes, insulin therapy is available when they do not respond to oral medications. Insulin therapy includes short-acting, medium- acting, and long-acting insulins, with the appropriate type selected based on the patient's condition.

### Development and effectiveness of novel drugs

#### SGLT2 inhibitor

SGLT2 inhibitors lower blood glucose levels by inhibiting glucose reabsorption in the kidneys and excreting excess glucose in the urine. SGLT2 inhibitors also have a weight-loss effect, contributing to weight management in diabetic patients. It has been reported that SGLT2 inhibitors have beneficial effects on patients with heart failure or kidney disease, even without diabetes^10)^.

#### GLP-1 receptor agonist

GLP-1 receptor agonists work by mimicking GLP- 1, a hormone secreted by the gut. They promote postprandial insulin secretion and suppress appetite. They also have a weight-loss effect and help diabetics manage their weight^11)^. The drug works with a weekly injection and improves patient adherence. A more potent drug called a dual agonist (GLP-1 and GIP agonist) is also available, and the development of a triple agonist is in progress in Japan. Recently, GLP-1 receptor agonists have been prescribed for cosmetic reasons to patients without diabetes or obesity, which leads to inadequate supply for diabetic patients.

### The latest technology for diabetes treatment

#### Continuous glucose monitoring (CGM)

The continuous glucose monitoring (CGM) system is a technology that monitors blood glucose levels in real-time. CGM involves placing a sensor under the skin that estimates blood glucose levels based on the glucose level in the interstitial fluid (the fluid surrounding the body's cells), allowing patients to intermittently monitor their blood glucose levels a whole day. This allows patients to respond quickly to sudden fluctuations in blood glucose levels, enabling more effective blood glucose management. The CGM can be linked to a smartphone so that patients can check their blood glucose data anytime. It can also share data with healthcare professionals, enabling remote medical care and advice.

### Insulin pump

An insulin pump is a device that continuously delivers insulin to the body. Insulin pumps can automatically adjust insulin doses in response to fluctuations in blood glucose levels, allowing for more precise management of blood glucose levels. Insulin pumps mimic natural insulin secretion by providing continuous basal insulin and additional insulin needed at mealtime. Modern insulin pumps, in conjunction with CGM, are expected to prevent hypoglycemia and hyperglycemia and keep patients healthy. The use of insulin pumps improves patients' quality of life and facilitates self-management^12)^. In recent years, automated insulin administration systems (artificial pancreas) have also been developed, with hybrid closed-loop systems combining CGM and insulin pumps. This system monitors blood glucose levels in real time and automatically adjusts insulin doses as needed. This minimizes fluctuations in blood glucose levels and provides ideal blood glucose control.

### Goals of diabetes treatment

**Figure 4:**
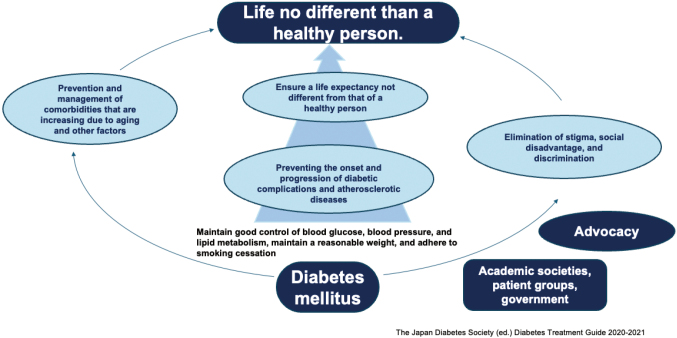
An aim for treatment of diabetes. The importance of eliminating stigma via advocacy is stressed

The goal of diabetes treatment is for the patient to lead a life similar to that of a healthy person (Figure 4)^13)^. To this end, proper glycemic control and prevention of complications are essential. To prevent complications, it is important to control blood glucose levels within the proper range.

#### Overcoming the stigma against diabetes

**Figure 5:**
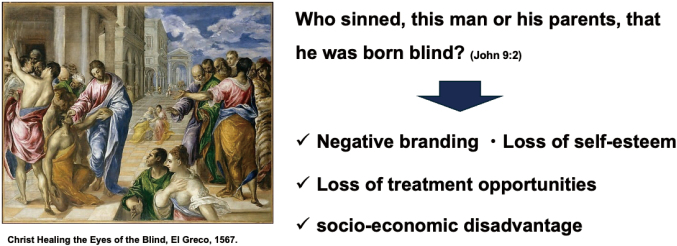
An example of stigma shown in the bible

Stigma against people with diabetes can undermine patients' self-esteem and discourage them from seeking treatment. Diabetes is a difficult disease to self-manage, and social misunderstanding and prejudice can discourage patients from seeking treatment (Figure 5). Society must deepen its understanding of diabetes through educational activities. Stigma can cause patients to develop negative feelings about self-management and reduce their motivation for treatment. Stigma faced by diabetics includes prejudices such as overeating and laziness. To dispel these prejudices, it is important to disseminate correct knowledge about diabetes, which includes advocacy by the groups associated with diabetes. For example, the Japanese Association for Diabetes Education and Care and The Japan Diabetes Society jointly proposed in 2023 that the disease name “*tonyo-byo*” should be renamed to “*diabetes*”, pronounced in a Japanese way.

### Importance of basic research

**Figure 6:**
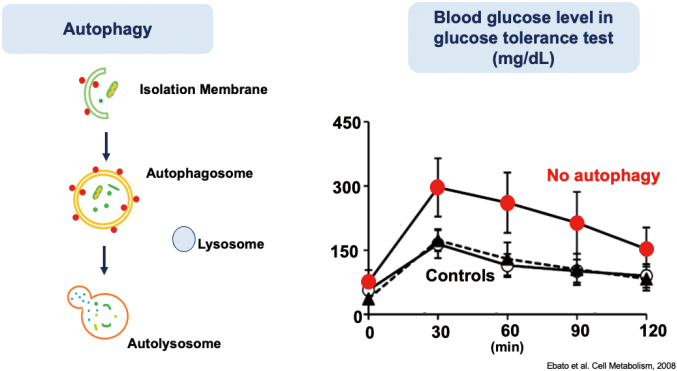
The importance of autophagy in pancreatic beta cells. Loss of autophagy in beta cells leads to glucose intolerance

Basic research is important to advance the treatment of diabetes. Interestingly, many important scientific discoveries are related to diabetes. To start with the discovery of insulin in 1921, the development of radio-immunoassay for measuring insulin and determining the amino acid sequence of insulin are noteworthy. In our department, research is underway to elucidate the relationship between autophagy (self-digestion) and beta cell function. Autophagy is a process that breaks down self-components in the cell and promotes the production of new components. We discovered that lack of autophagy in pancreatic beta cells induces glucose intolerance (Figure 6)^14)^. Elucidating how this process contributes to maintaining beta cell homeostasis could lead to developing new therapies that promote beta cell function. Our goal is to improve the quality of diabetes treatment by applying the results of basic research.

### Diabetes education program

Education and awareness of diabetic patients is essential to the effectiveness of treatment. In our educational inpatient program at Juntendo University Hospital, patients receive comprehensive education on diet, exercise, and medication. In the educational inpatient program, patients are hospitalized for approximately one week and receive comprehensive education, including

**1. Dietary education:** provide patients with an appropriate meal plan and explain the importance of a nutritionally balanced diet. The patient will learn how to implement this diet in their daily lives by providing specific dietary examples.

**2. Exercise Therapy Education:** Explain the types and appropriate amount of exercise and suggest an exercise program appropriate for the patient. Practice specific exercise methods, such as walking and strength training, to establish an exercise routine that is easy to incorporate into daily life.

**3. Medication education:** explain in detail the mechanism of action, side effects, and dosage of various medications. Patients are taught to understand and accurately follow their own medication regimen. Practice insulin injections and self-monitoring of blood glucose if needed.

### Effectiveness of educational programs

This allows patients to improve their self-management skills. Patients who undergo an educational inpatient program often have improved self-management skills and better glycemic control. In addition, as patients understand their disease and continue with appropriate treatment, the risk of complications is reduced and quality of life is improved.

## Conclusion

Diabetes is a treatable and manageable disease, but it requires the understanding and cooperation of society. It is important to continue to help diabetic patients lead better lives by utilizing the latest treatments and basic research findings. Overcoming the stigma against diabetes through advocacy and protecting patients' self-esteem is also essential to improving the effectiveness of treatment. Progress in diabetes care depends on the cooperation of healthcare professionals, patients, and society. We will continue our efforts to improve the quality of diabetes treatment by incorporating the latest research findings. By doing so, we aim to enable diabetes patients to lead lives no different from those of healthy people.

## Funding

No funding was received.

## Author contributions

YN wrote the draft. HW reviewed the manuscript. All authors read and approved the manuscripts.

## Conflicts of interest statement

The Authors declare that there are no conflicts of interest.
